# A single locus determines praziquantel response in *Schistosoma mansoni*

**DOI:** 10.1128/aac.01432-23

**Published:** 2024-01-30

**Authors:** Frédéric D. Chevalier, Winka Le Clec’h, Matthew Berriman, Timothy J.C. Anderson

**Affiliations:** 1 Host-Pathogen Interactions Program, Texas Biomedical Research Institute, San Antonio, Texas, USA; 2 School of Infection and Immunity, University of Glasgow, Glasgow, UK; 3 Disease Intervention and Prevention Program, Texas Biomedical Research Institute, San Antonio, Texas, USA; The Children's Hospital of Philadelphia, Philadelphia, USA

**Keywords:** genetic mapping, schistosome, drug resistance

## Abstract

We previously performed a genome-wide association study (GWAS) to identify the genetic basis of praziquantel (PZQ) response in schistosomes, identifying two quantitative trait loci situated on chromosomes 2 and 3. We reanalyzed this GWAS using the latest (version 10) genome assembly showing that a single locus on chromosome 3, rather than two independent loci, determines drug response. These results reveal that PZQ response is monogenic and demonstrates the importance of high-quality genomic information.

## INTRODUCTION

**Fig 1:**
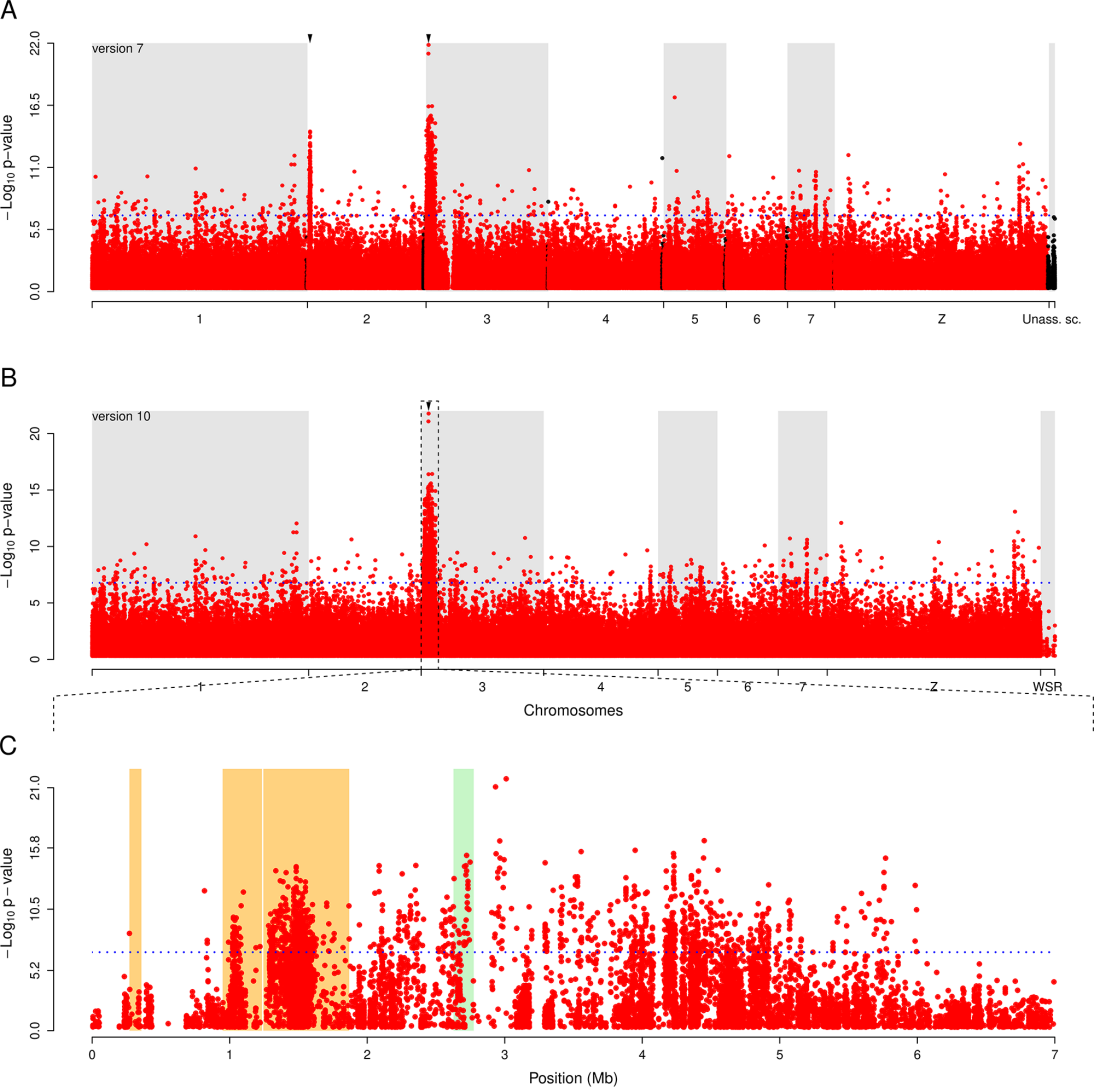
Comparison of the genome-wide association mapping of PZQ response against versions 7 and 10 of the *S. mansoni* reference genome. The Manhattan plot identifies genome regions that differ in allele frequency between PZQ-sensitive and PZQ-resistant worm pools. While the mapping done with the version 7 of the reference genome revealed two QTL peaks on chromosomes 2 and 3 (A), mapping against the version 10 of the genome highlighted a single peak on chromosome 3 (B). The QTL on chromosome 2 was actually an artifact due to misplaced sections of the chromosome 3 (orange box) (C). Blue dotted line refers to the Bonferroni significance threshold; red and black dots represent association of individual single nucleotide polymorphisms (SNPs) for assembled and unassembled scaffolds, respectively; green box marks the position of the *Sm.TPRM*_*PZQ*_ gene.

In a recent research article (1), we performed a genome-wide association study (GWAS) to identify the genetic basis of praziquantel (PZQ) response in schistosomes. This study leveraged a mixed population of PZQ-resistant and PZQ-sensitive *Schistosoma mansoni* generated by laboratory selection (SmLE-PZQ-R) (2), and we compared genome-wide allele frequencies in adult worms that recovered or failed to recover from PZQ treatment to determine the genetic basis of PZQ response. We identified two quantitative trait loci (QTL) associated with drug response situated on chromosomes 2 and 3 (Fig. 1A). On chromosome 3, we determined that the *Sm.TRPM_PZQ_* gene, which encodes a transient potential receptor channel, was the cause of variation in PZQ response. This conclusion was further supported by an independent study using pharmacological approaches (3). In addition, we showed lower *Sm.TRPM_PZQ_* gene and isoform expression in PZQ-resistant (PZQ-R) worms, suggesting that expression level may determine the PZQ response phenotype. However, we were unable to identify a causative gene within the chromosome 2 QTL. In this note, we revisit the published data set using an updated and improved genome assembly to further investigate the chromosome 2 QTL, to try to resolve the inconsistencies observed in Le Clec’h *et al*. (1), and to better understand the genetic architecture of PZQ response.

We used the version 7 of the *S. mansoni* genome to map our genomic data in Le Clec’h *et al*. (1). This version contained 312 unassembled scaffolds, many of which represented alternative haplotypes for regions across the genome, in addition to the near complete assembly of the seven autosomes and ZW sex chromosomes. However, the most recent of the *S. mansoni* reference genome assemblies [versions 9 (4) and 10] have been substantially improved. The latest version 10 of the genome [available on WormBase ParaSite (5)] is now assembled in full-length chromosomes. The unassembled scaffolds have been either incorporated into the main chromosomal assemblies or removed as haplotypic alternatives for many regions. Importantly, some chromosomal regions have been reassigned to their correct chromosomal locations (either on the same chromosome or on a different chromosome). These assembly changes have particularly affected not only the contiguity of the sex chromosomes but also the regions of chromosome 2. In parallel with the assembly revision, gene models have been further improved in version 10 to better reflect experimental evidence; predicted transcript isoforms are now only included when alternative intron:exon boundaries are supported by ≥10% alignment depth from a single RNA sequencing library. This gene model curation has resulted in a global reduction in transcript isoforms predicted: while 27.58% of gene models encoded more than one isoform in version 7, this number fell to 8.65% in version 10. The *Sm.TRPM_PZQ_* is one of the genes affected, with a reduction in number of major annotated isoforms from 7 to 3. With the release of this revised and improved reference genome, we took the opportunity to revisit our genetic mapping experiments to re-evaluate the genetic architecture of PZQ response in schistosomes. We also reanalyzed our transcriptomic data to determine whether *Sm.TRPM_PZQ_* gene expression remains associated with differences in PZQ response.

Our revised genetic mapping using the latest version of the *S. mansoni* reference genome revealed a single QTL on chromosome 3 associated with PZQ response. The chromosome 2 QTL is no longer present (Fig. 1B). This change is a direct consequence of the relocation of sections of chromosome 2 to chromosome 3 (Fig. 1C). This is reflected by the size of the new QTL (5,723,424 bp), which is approximately the sum of the two previous QTLs (chromosome 2: 1,166,271 bp; chromosome 3: 3,990,733 bp; total: 5,157,004 bp). The chromosome 2 QTL is now located, in three segments, on the boundary of the chromosome 3 QTL, near the beginning of the p-arm of the chromosome. The location on the boundary of the chromosome 3 QTL is likely to explain the absence of correlation between phenotype and genotype previously shown using individual worms (1). The relocation of chromosome 2 QTL increased the total number of genes under the updated chromosome 3 QTL from 91 to 137, of which 125 are expressed in adults (Table S1). The strongest associated SNP marker (position 3,010,821T > C) was still located in the *SOX13* transcription factor gene (*Smp_345310*). In addition, the two deletions previously identified close to the *Sm.TRPM_PZQ_* and the *SOX13* genes and associated with the resistant phenotype were confirmed by remapping to the version 10 genome (positions 2,775,001–2,900,000 and 3,175,001–3,300,000, respectively).

**Fig 2:**
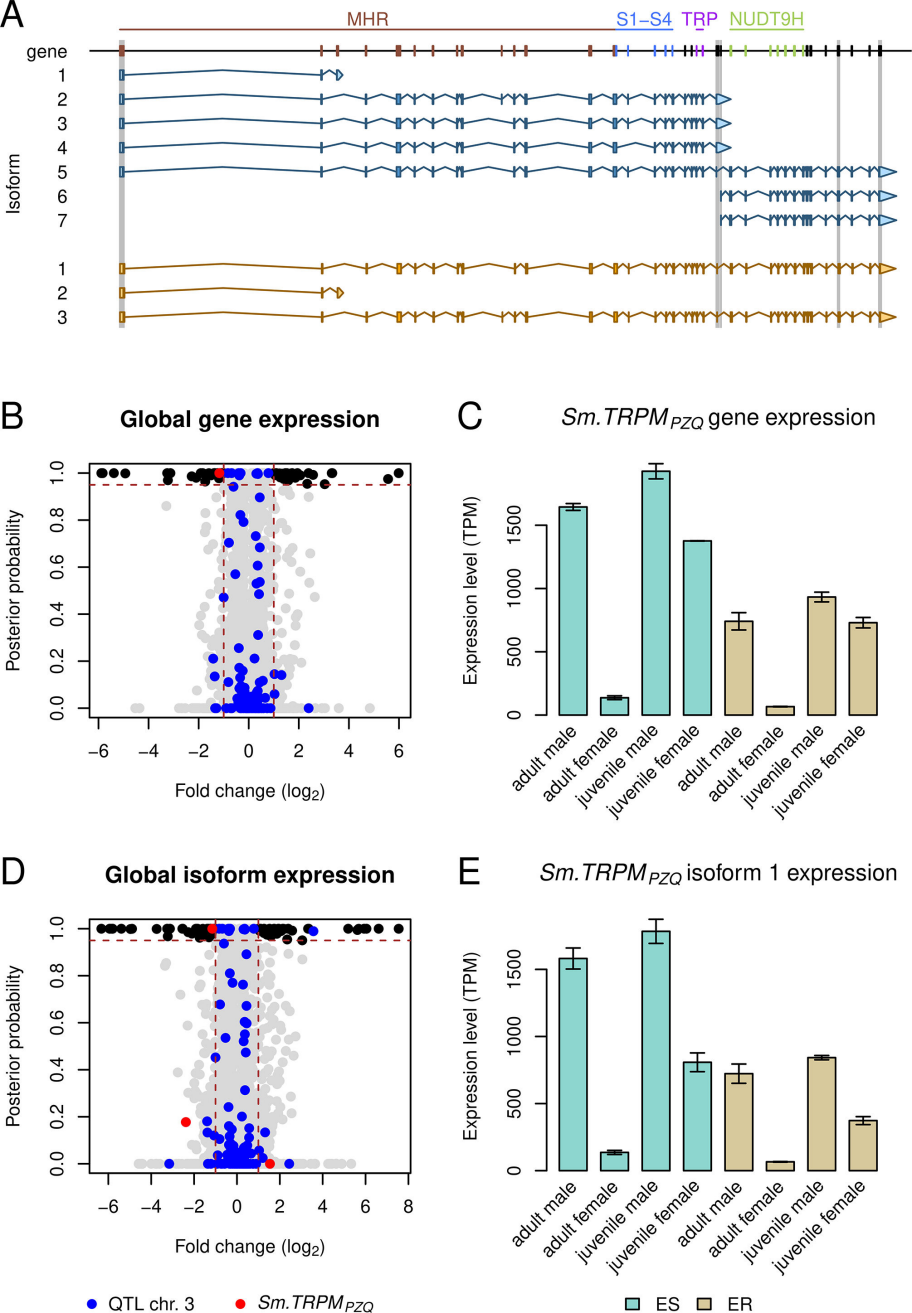
Gene expression differences between SmLE-PZQ-ES and SmLE-PZQ-ER parasites. (**A**) The gene and isoform annotations were revised with the version 10 of the reference genome, which led to an updated *Sm.TRPM_PZQ_* gene model. The number of predicted isoforms changed from 7 to 3: isoforms 3, 4, 6, and 7 were discarded; isoforms 2 (now 3) and 5 (now 1) were updated. Changes in exons (either removal or updates of their boundaries) are highlighted with gray boxes. Exons encoding major protein domains are highlighted on the gene model: the TRPM homology region (MHR) domain (brown), the four transmembrane-spanning helices (S1–S4) (blue) and TRP box (purple), which are key in the interaction between the channel and PZQ, and the NUDT9H domain (green). (**B**) The *Sm.TRPM_PZQ_* gene is the only gene under the chromosome 3 QTL to show differential expression between adult male PZQ-ES and PZQ-ER. (**C**) *Sm.TRPM_PZQ_* gene showed reduced expression in PZQ-ER parasites at all stages and for both sexes. Gene expression is overall higher in juveniles than adults, while gene expression in adult females is very low, likely explaining their natural resistance to PZQ. (**D**) Among the three isoforms of the *Sm.TRPM_PZQ_* gene, only isoform 1 showed differential expression. (**E**) The expression pattern of this isoform is very similar to the gene expression pattern, with high expression in naturally resistant juveniles. On the volcano plots, black dots show genes or isoforms with significant differential expression genome-wide, blue dots show genes or isoforms located under the chromosome 3 QTL, red dots show *Sm.TRPM_PZQ_* gene or isoforms, vertical brown lines show a twofold threshold in differential expression, horizontal brown line shows a threshold of 0.95 posterior probably in differential expression.

We revisited our transcriptomic analysis using the updated annotation produced alongside the version 10 of the reference genome. This updated annotation included a revised *Sm.TRPM_PZQ_* gene model with minor changes in number of exons but extensive alterations in number of isoforms (Fig. 2A). The total number of exons was reduced from 41 to 38, with modifications to the boundaries of some exons. As expected, these minor changes had a limited impact on the overall gene expression. Our revised transcriptomic analysis confirmed the reduced expression of *Sm.TRPM_PZQ_* gene in the SmLE-PZQ-ER parasites (enriched for PZQ-R allele) (Fig. 2B and C) in adult and juvenile worms of both sexes. We confirmed that the *Sm.TRPM_PZQ_* gene is the only gene under the QTL with a significant change in expression (Fig. 2B). The gene has a lower expression in SmLE-PZQ-ER adult male and female worms compared to their SmLE-PZQ-ES counterparts (Fig. 2C). We also confirmed very low expression in adult females of both SmLE-PZQ-ER and SmLE-PZQ-ES and the higher gene expression pattern in SmLE-PZQ-ES juvenile worms.

However, the updated analysis of isoform expression differed in several ways from that previously observed. The revised gene models resulted in reduction of annotated isoforms from 7 (version 7) to 3 (version 10) (Fig. 2A). Specifically, isoforms 3, 4, 6, and 7 from version 7 are no longer present. Isoform 1 is the most abundant of the three isoforms, accounting for the majority (88.32–100%) of *Sm.TRPM_PZQ_* transcripts in adults. We detected reduced expression of isoform 1 (formerly isoform 5) in SmLE-PZQ-ER adult males compared to SmLE-PZQ-ES adult males. SmLE-PZQ-ER adult males show comparable expression of isoform 1 to SmLE-PZQ-ES juvenile females that are naturally resistant. The new annotation of the version 10 genome does not exclude the possibility of rarer exon combinations, but the simplified isoform profile, and the predominance of isoform 1, now allows us to focus on this isoform for future analyses. We previously hypothesized that isoform 6 expression might be associated with PZQ sensitivity because of its high expression only in SmLE-PZQ-ES adult males (1). Isoform 6 corresponded to the terminal 15 exons of the gene model but, from existing short-read data, there is insufficient evidence to infer an appropriate transcriptional start for this truncated isoform. The isoform, therefore, no longer exists in the version 10 assembly and the association is likely to be spurious and allowing us to reject this hypothesis.

Our reanalysis now clearly shows that PZQ response is a single gene recessive trait in the laboratory populations studied and does not involve two independent loci as previously indicated (1). This is consistent with the observation that drug resistance typically has a simple genetic basis and is often monogenic (6). If PZQ resistance is also monogenic in natural parasite populations, this will greatly simplify molecular monitoring in control programs. Sequencing exons of *Sm.TRPM_PZQ_* from miracidia larvae or worm pools by either direct PCR amplification or targeted capture libraries will reveal possible PZQ resistance mutations and their frequencies. Putative resistance mutations could then be validated using *in vitro* assays (3). Reanalysis of the transcript data using version 10 annotation also simplifies our understanding of this system, showing that just isoform 1 (of three isoforms) predominates and shows differential expression between resistant and sensitive parasites. Future work can focus on how expression of isoform 1 impacts drug response. These updated results, utilizing the latest high-quality *S. mansoni* reference genome, underscore the importance of a robust reference genome for precise genetic mapping of critical biomedical traits, such as drug resistance in pathogens. Similar efforts to enhance genome assemblies have also been recently undertaken for the two other major schistosome species, *Schistosoma haematobium* and *Schistosoma japonicum* (7, 8). High-quality genomes have also improved our understanding of drug resistance in *Haemonchus contortus*, a major gastrointestinal nematode of small ruminants (9). Our present results serve as a compelling illustration of the ongoing need for continuous improvements in genome assembly, after the initial publication (10).

## Data Availability

Sequence data is available from: PRJNA699326, PRJNA701978, PRJNA704646. Code is available on Zenodo (DOIs: 10.5281/zenodo.10407184 and 10.5281/zenodo.10407187).

## SUPPLEMENTAL MATERIAL

Table S1 aac.01432-23-s0001.xlsx Genes under QTL of chromosome 3.

ASM does not own the copyrights to Supplemental Material that may be linked to, or accessed through, an article. The authors have granted ASM a non-exclusive, world-wide license to publish the Supplemental Material files. Please contact the corresponding author directly for reuse.
